# Implementation of sequencing-based surveillance for drug-resistant tuberculosis in Nigeria

**DOI:** 10.3389/ftubr.2026.1855903

**Published:** 2026-07-02

**Authors:** Heather P. McLaughlin, Israel Audu, Monday Tola, Chika Kingsley Onwuamah, Julius Ogwu, Kenneth Atoe, Chioma Nneka Kunle-Ope, Isiyaku Ahmadu, Joshua Gowon, David B. Meshak, Mosunmola Iwakun, Chinedu Ejianya Ude, Modupe Abiona, Abidemi Esther Momoh, Temiloluwa Ore, Chinenye Angela Ogbu, Temitope Abigail Abiodun, Jamie Dawson, Patricia Hall-Eidson, Nnaemeka C. Iriemenam, McPaul Okoye, Clement Adesigbin, Obioma Chijioke-Akaniro, Elom Emeka, Anyaike Chukwuma

**Affiliations:** 1 Division of Global HIV and TB, U.S. Centers for Disease Control and Prevention, Atlanta, GA, United States; 2 Division of Global HIV and TB, U.S. Centers for Disease Control and Prevention, Abuja, Nigeria; 3 Institute of Human Virology Nigeria, Abuja, Nigeria; 4 Department of Microbiology, Nigerian Institute of Medical Research, Lagos, Nigeria; 5 National Tuberculosis Reference Laboratory, Zaria, Nigeria; 6 National Tuberculosis and Leprosy Control Programme, Federal Ministry of Health, Abuja, Nigeria

**Keywords:** drug resistance, implementation, sequencing, surveillance, tuberculosis

## Abstract

Sequencing for drug-resistant tuberculosis (DR-TB) surveillance was established in Nigeria for the first time, demonstrating a nationally coordinated referral network for multi-disease sequencing services. *Mycobacterium tuberculosis* isolates previously identified as resistant to rifampicin and/or isoniazid by genotypic or phenotypic drug susceptibility testing were selected proportionately across all six geopolitical zones in Nigeria. Programmatic use of sequence data for DR-TB surveillance was prioritized, demonstrating additional resistance detection and enhanced monitoring as new TB treatment regimens are scaled up. Sanger sequencing identified resistance to rifampicin and isoniazid missed by drug susceptibility tests routinely used in country, for 11% (5/47) and 2% (1/47) of isolates, respectively. This pilot study demonstrates feasibility and provides an example for programmatic implementation of integrated sequencing, supporting country efforts to strengthen genomic surveillance for DR-TB and improve prevention and control globally.

## Introduction

Globally, it is estimated that 13% of deaths attributed to antimicrobial resistance are due to drug-resistant tuberculosis (DR-TB), much of which remains undetected (1). In 2024, the number of people who developed rifampicin-resistant (RIF-R) and multidrug-resistant TB (MDR-TB), defined as resistant to both rifampicin (RIF) and isoniazid (INH), was estimated to be 390,000 (2). Culture-based phenotypic drug susceptibility testing (pDST) has been the gold standard for decades; however, this method is limited by inaccuracy in detecting particular cases of resistance to the critical first-line anti-TB drug RIF (1). Genotypic drug susceptibility testing (gDST) has accelerated turnaround time to bacteriological-confirmation of DR-TB but is restricted to interrogating narrow regions within a limited number of *Mycobacterium tuberculosis* (Mtb) resistance-associated genes (3). Missed or erroneous detection of drug resistance can lead to gaps in DR-TB surveillance and mismanagement of treatment, perpetuating TB transmission and driving the epidemic. Sequencing-based detection of DR-TB can offer a more comprehensive approach to surveillance and is included in global guidance for programmatic implementation (4), however, practical barriers related to cost, laboratory infrastructure, and technical expertise may hinder introduction.

Addressing DR-TB has been prioritized globally, including in Nigeria, a high HIV/TB- and multidrug-resistant (MDR)-TB burden country. As part of Nigeria's National Strategic Plan, 2021–2025 (5), the National Tuberculosis and Leprosy Control Program (NTBLCP) aims to enhance surveillance and management of DR-TB. Successful implementation of molecular surveillance for DR-TB, including sequencing, can contribute to effective monitoring, guide country policies and treatment strategies based on drug-resistance patterns, and be used to assess the impact of disease control interventions. Sequencing can address limitations of genotypic and phenotypic DSTs and complement results by providing a more complete picture of drug resistance. Moreover, an integrated, multi-disease sequencing approach can address challenges associated with implementation such as limited resources. Herein, we describe the establishment of Nigeria's first sequencing model for DR-TB surveillance in 2022, a programmatic introduction of services into existing sequencing laboratories previously established for other diseases.

## Design, findings, and discussion

Nigeria conducts TB culture and DST as part of the nation's diagnostic algorithm to detect and manage DR-TB. Clinical sputum specimens received by zonal and national TB reference laboratories (NTRLs) from health facilities across Nigeria are initially subjected to globally-recommended gDSTs. These tests include Xpert MTB/RIF (Cepheid), for TB diagnosis and detection of RIF resistance, followed by the first- and second-line Line Probe Assays [LPAs; GenoType MTBDR*plus*v2 and GenoType MTBDR*sl*v2 (Hain Life Sciences)], for detection of resistance to RIF, INH, fluoroquinolones (FQs), and second-line injectables (SLIs). Drug-resistant isolates are referred to NTRLs for subsequent pDST to the drugs listed above and to new and repurposed drugs used to treat DR-TB, including bedaquiline (BDQ) and clofazimine (CFZ). The automated BACTEC MGIT 960 system (MGIT) (Becton, Dickinson and Company) is used to perform pDST with globally-recommended drug concentrations (6).

**Figure 1:**
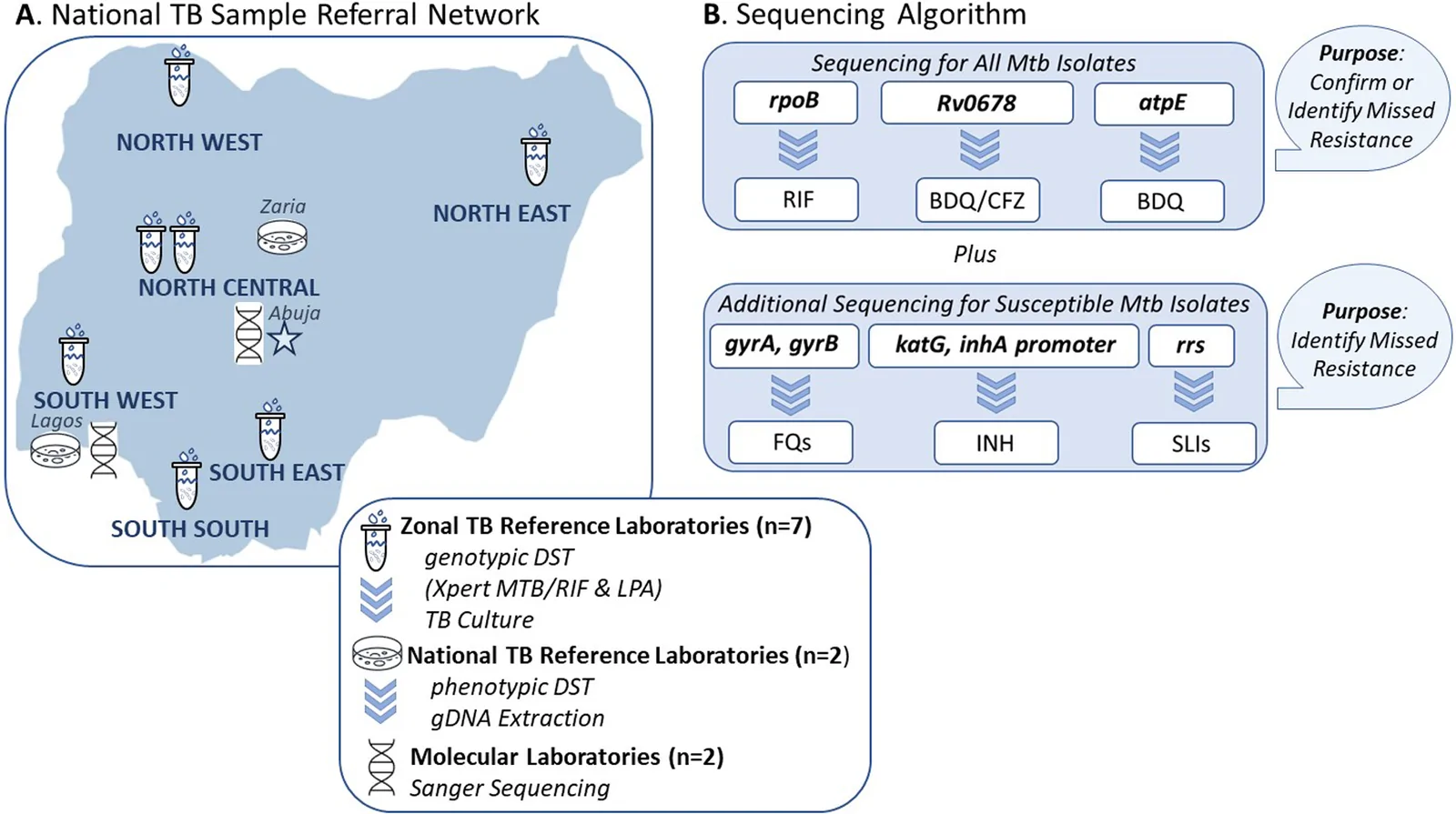
Nigeria's first hub-and-spoke sequencing network for drug-resistant TB surveillance. A national hub-and-spoke DR-TB sequencing network and algorithm were established in Nigeria for the first time **(A)** Clinical sputa collected and confirmed positive for TB at seven zonal TB reference laboratories from all six geopolitical zones were subjected to genotypic Drug Susceptibility Testing (DST) with Xpert MTB/RIF, first-, and second-line Line Probe Assays (LPAs). Samples were cultured on Lowenstein Jensen (LJ) medium and referred to two national TB reference laboratories for MGIT-based phenotypic DST and genomic DNA (gDNA) extraction. Extracted gDNA was then sent to two Sanger sequencing laboratories previously used only for HIV. Federal Capital Territory (star). A Sanger sequencing algorithm was purposefully designed **(B)** so that all *M. tuberculosis* (Mtb) isolates were sequenced to confirm or identify missed resistance to rifampicin (RIF), bedaquiline (BDQ), and clofazimine (CFZ) (top panel). All isolates that were susceptible to isoniazid (INH, *n* = 19), fluoroquinolones (FQ, *n* = 41) or second-line injectables (SLI, *n* = 44) were sequenced to identify mutations that were either not targeted by LPA or missed (bottom panel).

With support from the U.S. President's Emergency Plan for AIDS Relief (PEPFAR), Nigeria established an HIV drug-resistance sequencing program. Through the current work, the U.S. Centers for Disease Control and Prevention (CDC) built on this to establish the first hub-and-spoke sequencing network for DR-TB surveillance. Under NTBLCP leadership, national programmatic priorities informed strategies for isolate selection based on resistance profiles, sample referral, and Sanger sequencing algorithm design (Figure 1). *M. tuberculosis* isolates (*n* = 49) previously identified as drug-resistant to RIF and/or INH by LPA or MGIT were selected proportionately across all six geopolitical zones of Nigeria and considered for sequencing. For efficient introduction of sequencing for DR-TB, NTRLs were linked with existing HIV-DR sequencing laboratories, integrating sequencing services for the first time for these pathogens (Figure 1A). Inactivation of Mtb cultures and gDNA extractions were performed at NTRLs using heat (95 °C, 20 min) and ultrasonication (15 min). A courier-based referral pathway was established to ship genomic DNA (gDNA) to sequencing laboratories. Samples were assessed for quality and quantity using a NanoDrop spectrophotometer (Thermo Scientific) and a Qubit fluorometer (Invitrogen). As part of a purposefully designed algorithm, sequencing of eight genomic targets associated with resistance to six anti-TB drugs was completed to maximize programmatic use of data while ensuring cost efficiency (Figure 1B). *rpoB*, associated with resistance to RIF, was sequenced for all Mtb isolates to confirm or identify resistance missed by other DSTs as well as help settle instances of discordance between tests. *Rv0678* and *atpE*, associated with resistance BDQ and CFZ, were also sequenced for all isolates to introduce molecular testing for these newer drugs and to compare results with pDST. Only isolates determined to be drug susceptible to FQs, INH, and SLIs by LPA and MGIT were selected for sequencing *gyrA* and *gyrB*, *katG* and the *inhA* promoter, and *rrs*, respectively, to identify mutations not targeted by LPA and detect missed resistance by either test. Sanger sequencing was performed using an Applied Biosystems 3130xL or 3500xL Genetic Analyzer (Life Technologies) with previously published primers (7, 8) and the Terminator v 3.1 Cycle Sequencing Kit (Applied Biosystems). Bioinformatic analyses were performed using the software solution BacterioChek®-TB (Advanced Biological Laboratories) and Geneious Prime (v2022.2.2) whereby identified mutations were subsequently cross-referenced with the global catalogue of mutations (9) to determine resistance association. Additional details related to methodology, sampling, and validation testing may be found in Supplemental File 1.

**Figure 2:**
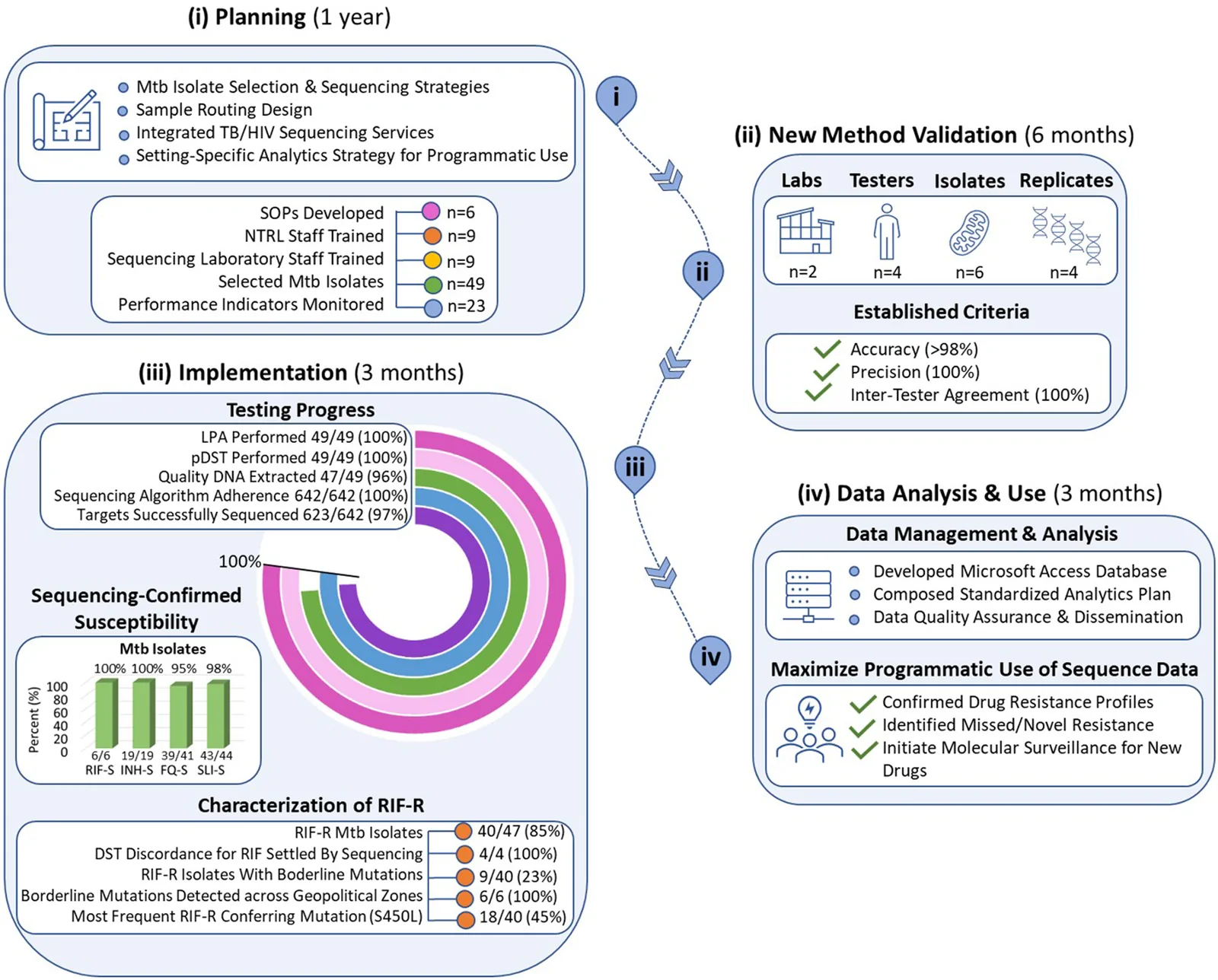
A phased approach for the programmatic introduction of sequencing-based drug-resistant TB surveillance in Nigeria. The introduction of sequencing-based DR-TB surveillance included four phases: (i) planning, (ii) new method validation, (iii) implementation, and (iv) data analysis for programmatic use. *M. tuberculosis* (Mtb), Standard Operating Procedure (SOP), National TB Reference Laboratory (NTRL), Line Probe Assays (LPA), phenotypic drug susceptibility testing (pDST), rifampicin (RIF), isoniazid (INH), fluoroquinolones (FQs), and second-line injectables (SLIs), resistant (R), susceptible (S).

The national introduction of sequencing-based DR-TB surveillance included four phases carried out over two years: (i) planning, (ii) new method validation, (iii) implementation, and (iv) data analyses for programmatic use (Figure 2). Planning for the implementation of quality sequencing services involved building technical capacity of the TB program and laboratory staff through training and developing standard operating procedures and new sequencing-specific quality control and performance indicators. New method validation of the sequencing process, from gDNA extraction, inactivation verification of extracted products, and interpretation of results, was successfully conducted at NTRLs and sequencing laboratories, with all laboratories exceeding established acceptance criteria. Of the 49 drug-resistant Mtb isolates selected for sequencing, all were tested by gDST (LPA) and pDST. Genomic DNA suitable for downstream sequencing was successfully extracted from 96% (47/49) of isolates, 100% adherence to the sequencing algorithm was achieved, and high-quality results for algorithm-determined loci were obtained for 623 (97%) of 642 sequencing reactions (Figure 2). A Microsoft Access database was created to manage data. A standardized analytics plan was developed to investigate genomic mutations against upstream DST results and maximize the programmatic use of sequencing data for DR-TB surveillance.

While sequencing analyses largely confirmed resistance reported by DSTs, it identified additional resistance to RIF for 5 (11%) and to INH for 1 (2%) of 47 isolates, initially missed by pDST and/or LPA (Table 1). Discordance was observed between LPA and pDST results for RIF for 4 (9%), INH for 7 (15%), FQs for 4 (9%), and SLIs for 3 (6%) of 47 Mtb isolates. Based on the conditional algorithm design, sequencing could be used to settle all four instances of discordance between DSTs for RIF, confirming *rpoB* mutations in three isolates determined to be resistance by LPA and one by pDST. Concordance between Xpert MTB/RIF and sequencing to detect resistance to RIF was observed for these four isolates as well as NIMR/E/59 in which LPA and MGIT both determined to be susceptible. One instance of discordance was recorded for isolate NIMR/A/03 in which LPA and pDST methods predicted RIF-R but no mutation was observed in *rpoB* by sequencing. No mutations were detected outside of the 81 bp rifampicin resistance determining region (RRDR) in *rpoB* which has been widely associated with RIF-R and no *rpoB* sequences contained more than one mutation.

**Table 1:** Sequencing-based detection of resistance to rifampicin and isoniazid, initially missed by genotypic (line probe assay) and phenotypic (MGIT) drug susceptibility tests routinely used in Nigeria.

*Mtb* isolate	LPA (gDST)	MGIT (pDST)	Sanger sequencing	*Gene* (mutation detected)		Missed drug resistance
				Manual analysis	Automated software	
NIMR/E/46	RIF-S	RIF-R	RIF-R	*rpoB*, L452P **(Borderline)**	*rpoB*, L452P **(Borderline)**	INH-R ► MDR (LPA vs. Sequencing)
NTBRL/C/02	RIF-R	RIF-S	RIF-R	*rpoB*, L452P **(Borderline)**	*rpoB*, L452 **(Borderline)**	S ►RIF-R (MGIT vs. Sequencing)
NTBRL/A/23	RIF-R	RIF-S	RIF-R	*rpoB*, L430P **(Borderline)**	*rpoB*, L430P **(Borderline)**	S ►RIF-R (MGIT vs. Sequencing)
NTBRL/A/12	RIF-R	RIF-S	RIF-R	*rpoB*, S450L	*rpoB*, S450L	S ►RIF-R (MGIT vs. Sequencing)
NIMR/E/59	RIF-S	RIF-S	RIF-R	*rpoB*, H445L	*rpoB*, H445L	INH-R ► MDR (LPA & MGIT vs. Sequencing)
NTBRL/A/37	RIF-R	RIF-R	RIF-R	*rpoB,* ins_ttc, 1,300–1,303nt	Not detected	N/A
NTBRL-I-26	RIF-R	RIF-R	RIF-R	*rpoB,* S450L (Heteroresistance*)	Not detected	N/A
NTBRL/J/01	RIF-R	RIF-R	RIF-R	*rpoB,* del_aac, 1,308–1,310nt	Not detected	N/A
NTBRL/A/14	INH-S	INH-R	INH-R	*inhA* promoter, c-15t	*inhA* promoter c-15t	RIF-R ► MDR (LPA vs. Sequencing)
**Total no. missed resistance**	**RIF, *n* = 2** **INH, *n* = 1**	**RIF, *n* = 4** **INH, *n* = 0**	–	–	**RIF, *n* = 3** **INH, *n* = 0**	–

Sequencing-based prediction of rifampicin (RIF) and isoniazid (INH) resistance among Mtb isolates (*n* = 47), missed by genotypic DST [MTBDR*plus* Ver 2.0, Line Probe Assay (LPA)], phenotypic DST (BACTEC MGIT 960), and/or automated bioinformatics software (Bacterio-Check). The last row indicates the total number of Mtb isolates in which RIF-R or INH-R was missed by LPA, MGIT, or automated software. (*) Potential instance of RIF heteroresistance, indicated by a double chromatogram peak. Susceptible (S), Resistant (R), Multidrug-Resistant (MDR), insertion (ins), deletion (del).

Borderline mutations in *rpoB* associated with low-level resistance to RIF were identified by sequencing in nine isolates originating from all geopolitical zones (Figure 2). Phenotypic DST by MGIT can fail to detect these clinically relevant variants which may fuel transmission of resistance, if undiagnosed (10). In this study, pDST missed RIF resistance in two isolates with confirmed borderline mutations, L452P and L430P (Table 1). These results are consistent with other comparative studies on DST in high-burden settings (11) and underscore the potential of sequencing to detect resistance not captured by other drug susceptibility tests routinely used in country as well as support its integration into testing algorithms for surveillance. One potential instance of RIF heteroresistance, indicated by a double chromatogram peak, and a unique insertion mutation in *rpoB* were missed using automated bioinformatic software, which are often restricted to a database of defined mutations for resistance prediction.

No additional resistance was detected by sequencing *gyrA*, *gryB,* or *rrs* in Mtb isolates determined to be susceptible to FQs (*n* = 41) or SLIs (*n* = 44) by LPA or pDST. Despite a small number of pDST-determined BDQ- (*n* = 6) and CFZ-resistant (*n* = 3) Mtb isolates, no mutations were detected in the resistance-associated genes *Rv0678* or *atpE*, highlighting the importance of continued phenotypic testing for new drugs where molecular mechanisms and mutations associated with resistance are not yet fully understood (12).

Upon analyses completion, the NTBLCP convened a stakeholder's meeting to review strengths, challenges, lessons learned and intended programmatic uses of sequencing for DR-TB. Ensuring a strong laboratory quality management system and developing a monitoring and evaluation plan supported quality-assured testing, proactively addressed obstacles associated with new test introduction, and helped track progress of implementation and impact. Leveraging existing laboratory infrastructure and workforce expertise along with strategic design of the sequencing algorithm supported cost-effective and efficient implementation. Based on the successful phased introduction of TB services into existing sequencing investments in Nigeria and the added value of sequencing results that complemented the program's routine DST data, the NTBLCP is building on this integrated sequencing pilot project and embracing advances in technology by shifting from Sanger sequencing for DR-TB at HIV laboratories to targeted next-generation sequencing (tNGS) for more comprehensive detection of DR-TB at the multi-disease testing laboratory, Nigeria Centre for Disease Control and Prevention, to further improve surveillance and future clinical management. For strategic planning to advance DR-TB surveillance in Nigeria, strengths, opportunities, aspirations, and results associated with implementation of genomic sequencing are presented in a SOAR analysis (Table 2).

**Table 2:** SOAR analysis for using genomic sequencing to strengthen drug-resistant TB surveillance in Nigeria.

Strengths	Opportunities
Detect resistance to many anti-TB drug in a single test and identify resistance missed other DST methods Rule-in resistance to new and repurposed drugs used to treat DR-TB and investigate resistance to other drugs, not routinely tested for a part of a national algorithm Serves as a model for the introduction of sequencing for DR-TB surveillance in Nigeria and other high-burden settings Demonstrated capacity built, sample referral network establishment, processes validated, and programmatic uses for sequencing data that align with NTPCLP goals Integrated, multi-disease sequencing promoted cost savings and cross-capacity building Challenges encountered can be applied as lessons learned for scaling sequencing activities	A strong initial investment in sequencing method validation is vital to ensure quality testing and streamlined implementation of NGS Objectives should guide sample selection strategies and incorporation of sequencing into diagnostic algorithms to maximize programmatic use Data analytic plans aligned with programmatic goals for DR-TB management and integration into existing surveillance data systems will support sustainability Global guidance documents are available to support country adoption of NGS for DR-TB detection
Aspirations	Results
Build on the demonstrated utility of genomic sequencing by implementing NGS for a more comprehensive, high-throughput approach for resistance prediction Define a national framework to integrate sequencing into routine testing and surveillance programs in Nigeria Develop a data management strategy that supports the interpretation and use of NGS data for programmatic action Prepare to adapt TB sequencing services for clinical use in the future Strengthen national capacity for cross-pathogen genomic surveillance, in alignment with Africa CDC’s vision to enhance detection and response to disease outbreaks	Define the use (immediate and future) of NGS surveillance of DR-TB within Nigeria’s National Strategic Plan for TB control Establish an NGS technical working group to lead preparations, develop a budgeted operational plan, and oversee regulatory compliance Execute an efficient and cost-effective strategy for programmatic surveillance of DR-TB Establish a comprehensive quality assurance program and external quality assurance mechanism to support high-quality implementation of NGS Implement tNGS, globally recommended for detection of DR-TB, first for surveillance purposes building a foundation for clinical use in the future

Strengths, opportunities, aspirations, and results (SOAR) strategic planning framework for utilizing genomic sequencing for enhanced DR-TB surveillance in Nigeria. This forward-focused SOAR analysis concentrates on Nigeria's strengths, available opportunities, future aspirations, and desired results in-country for the implementation of sequencing-based DR-TB detection.

NGS can offer higher throughput testing and improved sensitivity to detect heteroresistance at lower frequencies compared to Sanger sequencing. Moreover, commercially-available, end-to-end tNGS solutions that include automated bioinformatic pipelines may expand test uptake and global guidance documents are available to support country adoption (13–15). This work supports integration of sequencing into surveillance frameworks, especially in high DR-TB burden settings, for enhanced detection and response. Limitations to this work include sample size and algorithm constraints that restrict generalizability and scope of sequencing results for the drug-resistant Mtb isolates tested. In addition, the lack of repeat phenotypic testing due to resource restrictions may have helped to resolve discordance observed between DST methods that known differences between molecular and culture-based testing could not explain.

## Conclusions

DR-TB sequencing-based surveillance services were introduced in Nigeria for the first time using a stepwise model that successfully leveraged existing resources to provide multi-disease services. Sequencing-based DST for existing, new, and repurposed TB drugs confirmed most resistance results and identified missed drug resistance by routine testing methods, thus demonstrating utility for improved routine DR-TB surveillance. This work demonstrates feasibility and provides plans for operationalization of DR-TB sequencing in a national program setting, which may serve as an example to other countries on their journeys to strengthen genomic surveillance. The current transition to globally recommended tNGS will continue to strengthen program awareness of the national distribution of circulating DR-TB strains and TB control efforts, enhance anti-TB drug selection and treatment for TB patients, and help guide programmatic testing policies.

## Data Availability

Data is available upon request from the corresponding author.
