# Supplementary material for: Implementation of sequencing-based surveillance for drug-resistant tuberculosis in Nigeria
Source: Front Tuberc. 2026 Jul 2;4:1855903. doi: 10.3389/ftubr.2026.1855903 (PMC13491420; doi:10.3389/ftubr.2026.1855903)
Supplement: Supplementary file 1 [file Supplementaryfile1.docx]

**Supplemental File 1**

**Methods**

*Mycobacterial culture*

Clinical specimens were decontaminated using the modified Petroff’s method (N- acetyl-L-cysteine and sodium hydroxide) and sediments were suspended in phosphate buffer solution (PBS). Supernatant was discarded after centrifugation (3000g x 4°C x 15min) and sediments were resuspended in PBS ahead of inoculation into Lowenstein Jensen (LJ) media at 37°C.

*Inactivation of M. tuberculosis culture, genomic DNA (gDNA) extraction and assessment*

A crude method for inactivation and preparation of gDNA was performed from solid or liquid Mtb culture. Either a 1 µl loopful of solid culture or a bacterial pellet obtained from centrifugation of 1 ml of liquid culture at 13,000g for 15 min was resuspended in 300 µl molecular grade water by vortexing. Mtb was inactivated in a 95°C water bath for 20 min, cells were lysed using an ultrasonic bath for 15 min, and cell debris was pelleted by centrifugation at 13,000g for 5 min. 200 µl of supernatant containing gDNA was removed by pipet. gDNA samples were confirmed to be inactivated by inoculating 100 µl onto LJ slants and monitoring for lack growth over eight weeks at 37°C. gDNA samples were assessed for quality and quantity using either a Nanodrop spectrophotometer and a Qubit fluorometer, respectively, and stored at -20°C. Quality controls steps for shipment and receipt of DNA samples at molecular laboratories included a minimum quantity of 0.1 ng/ul and maintenance of cold-chain. All samples were shipped by trained personnel and packaged according to national regulations.

*Phenotypic Drug Susceptibility Testing (pDST)*

The automated BACTEC MGIT 960 TB system was used for liquid phenotypic susceptibility testing of rifampicin (RIF), isoniazid (INH), fluroquinolones (FQs) moxifloxacin (MFX) and levofloxacin (LVX), second-line injectables (SLI) amikacin (AMK), kanamycin (KAN), and capreomycin (CAP), bedaquiline (BDQ), and clofazimine (CFZ). pDST was performed according to manufacturer’s instructions and globally-recommended critical concentrations.

*Genotypic Drug Susceptibility Testing (gDST)*

Rapid genotypic detection of drug resistance was performed using the globally recommended tests GeneXpert MTB/RIF (Cepheid) and first- and second-line Line Probe Assays (LPA) GenoType MTBDRplus V2 and GenoType MTBDRsl V2 (Hain Life Science). These molecular tests operate by detecting mutations most frequently identified in RIF-, INH-, FQ- and/or SLI-resistant M. tuberculosis (Mtb) isolates and these assays were performed according to manufacturer’s instructions. For LPAs, 5 µl of gDNA was used for PCR amplification, all of which were performed under the same PCR conditions.

*Gene amplification and Sanger sequencing*

Genes associated with resistance to RIF (*rpoB*), INH (*inhA* promoter, *KatG*), fluoroquinolones MFX and LVX (*gyrA, gyrB*), and second-line injectables AMK, KAN, CAP (rrs) were amplified using primers described in Rowneki *et al*. (7) and those associated BDQ and CFZ resistance (*atpE, Rv0678*) were amplified using primers outlined by Yang *et al*. (8). For quality control, each PCR product was confirmed by gel electrophoresis, ExoSAP-IT (ThermoFisher) was used to hydrolyze any remaining primers and unincorporated nucleotides in amplification reactions, and DNA cycle sequencing was performed using the Big Dye® Terminator v 3.1 cycle sequencing kit (ThermoFisher). Gene sequences were generated using the primers previously described, reactions were purified using the BigDye® XTerminator Purification kit, and DNA was sequenced on an Applied Biosystems 3130xL, 3500xL Genetic Analyzer (Life Technologies).

*Sequence-based resistance prediction*

Automated detection of mutations known to be associated with drug resistance was accomplished using BacterioChek-TB Software (Advanced Biological Laboratories). Briefly, AB1 sequence files were uploaded to the web-based application and sequenced loci were selected for analysis. Interpretation of drug-resistance was based on identification of high-confidence, resistance-associated mutations initially determined using the ReSeq algorithm, and more recently an updated algorithm based on the 2021 Global Catalogue of Mutations (9). Manual examination of Sanger sequencing chromatograms was also performed to identify additional or novel mutations within targeted loci using Geneious Prime v2022.2.2. The *M. tuberculosis* H37Rv genome (Genbank Accession Number NC_000962.3) was used as a reference for sequence alignment and identification of mutations required coverage by both forward and reverse primer reads. Mutations were subsequently cross-referenced with the Global Catalogue of Mutations or published literature to determine association or potential association with drug resistance.

*Sampling*

Mtb isolates (n=49) were selected proportionately across all six geopolitical zones (eight isolates from five zones and nine from the North Central zone). Isolates were considered for sequencing if they were previously identified as resistant to any first-line drug by LPA or pDST. Isolates with various drug-resistance patterns were investigated as part of this pilot project including those mono-resistant to RIF (n=16) or INH (n=5), multi-drug resistant (n=11), pre-extensively-drug resistant (n=4), and poly-drug resistant (n=13).

*Validation testing*

A method validation plan for drug-resistant TB Sanger sequencing was developed and successfully completed at both molecular laboratories at the Institute of Human Virology Nigeria (IHVN) and the Nigerian Institute of Medical Research (NIMR). Validation testing data provided evidence that Sanger sequencing of *M. tuberculosis* using the ABI 3500xL Genetic Analyzer and primers targeting genes associated with drug resistance produced results that met established acceptance criteria when performed according to manufacturer instructions and SOPs. Briefly, validation was completed using six Mtb isolates from an External Quality Assurance panel received from a TB Supranational Laboratory. These well-characterized isolates had known phenotypic and genotypic drug susceptibility testing profiles, including the presence or absence of mutations associated with resistance, as determined by analysis of whole-genome sequencing data. Two trained laboratorians at each molecular laboratory performed Sanger sequencing of all eight genes of interest for each of the six Mtb isolates, in duplicate and blinded to results. Sequence data was analyzed manually using bioinformatic tools and automatically using BacterioChek-TB software. Sequencing results were evaluated and acceptance criteria for accuracy, precision, and inter-tester agreement were successfully met by both molecular laboratories (100% concordance for drug resistance profiles and ≥98% concordance for mutation detection compared to expected results).
